# Complementary Split-Ring Resonator-Loaded Microfluidic Ethanol Chemical Sensor

**DOI:** 10.3390/s16111802

**Published:** 2016-10-28

**Authors:** Ahmed Salim, Sungjoon Lim

**Affiliations:** School of Electrical and Electronics Engineering, College of Engineering, Chung-Ang University, 221 Heukseok-Dong, Dongjak-Gu, Seoul 156-756, Korea; ahmedsalim789@gmail.com

**Keywords:** ethanol sensor, microfluidic channel, complementary split ring resonator (CSRR), patch

## Abstract

In this paper, a complementary split-ring resonator (CSRR)-loaded patch is proposed as a microfluidic ethanol chemical sensor. The primary objective of this chemical sensor is to detect ethanol’s concentration. First, two tightly coupled concentric CSRRs loaded on a patch are realized on a Rogers RT/Duroid 5870 substrate, and then a microfluidic channel engraved on polydimethylsiloxane (PDMS) is integrated for ethanol chemical sensor applications. The resonant frequency of the structure before loading the microfluidic channel is 4.72 GHz. After loading the microfluidic channel, the 550 MHz shift in the resonant frequency is ascribed to the dielectric perturbation phenomenon when the ethanol concentration is varied from 0% to 100%. In order to assess the sensitivity range of our proposed sensor, various concentrations of ethanol are tested and analyzed. Our proposed sensor exhibits repeatability and successfully detects 10% ethanol as verified by the measurement set-up. It has created headway to a miniaturized, non-contact, low-cost, reliable, reusable, and easily fabricated design using extremely small liquid volumes.

## 1. Introduction

Ethanol finds its numerous usages in the pharmaceutical, medical diagnosis, academic, and beverage industries to name a few. In such environments, there must be low-cost, miniaturized, potentially safe, and highly sensitive sensor devices working at ambient temperature to accurately analyze and monitor the ethanol concentration. Several highly sensitive sensors to detect ethanol have been proposed to date in the literature. However, a number of adverse factors, such as large size, high cost, potential lack of safety, complex design and dependency on temperature or other supportive conditions, decrease their efficacy.

State-of-the-art sensors, for example electrochemical sensors, optical sensors, and pure chemical sensors, exhibit excellent sensitivity. In electrochemical sensors, the resistance of a metal oxide semiconductor (MOS) sensor changes when gas molecules are adsorbed [1,2,3,4,5]. The change in analyte concentration is used to characterize the resultant current. Although MOS sensors exhibit excellent sensitivity, they suffer from a sparking risk at electrical connections and elevated temperature that gives rise to high power consumption [2]. The high temperature issue has been solved by modifying metal oxide nanoparticles using some noble metals such as Au [6] and Pd [7]; however, that makes these sensors uneconomical. Optical/photo sensors utilize receptors/sites on the sensor surface that are sensitive to a particular wavelength when illuminated by an optical source and thus provide information in terms of wavelength shifting, spectral variation, color change, or light intensity deviation. These sensors compensate the deficiencies of MOS sensors to some extent, as they can be operated at ambient temperature, while eliminating the need for electrical connections [8]. However, because of their bulky size they are not readily available to be integrated in miniaturized systems [8,9].

The integration of microfluidic channels with radio frequency (RF) components has led to an extremely useful field, where a microfluidic channel, filled with very small volumes of liquid, controls the resonant frequency of the structure [10]. Screening of multiple fluids simultaneously [11], DNA analysis [12,13], characterization of cell behavior in the gigahertz range [14], dielectric characterization [15], sensing of chemicals [16], and analysis of biomaterials [17] are intensively investigated applications of microfluidics. Especially, RF sensors integrated with microfluidics provide the benefits of non-contact, low cost, reusability and simple fabrication.

Metamaterial structures such as split-ring resonators (SRRs) and CSRRs have been used to realize microwave resonators, filters, and microwave antennas. The structure of the SRR/CSRR can be understood and analyzed by modeling the equivalent LC (inductance and capacitance) and applying the resonance phenomenon. There is a growing trend to realize communication and sensing applications using SRRs and CSRRs owing to their high quality (Q) factor [18,19,20,21,22,23,24,25,26,27,28,29,30]. The CSRR consists of a single ring, etched on the top metallization or in the ground plane with a particular orientation. Their integration with other structures significantly reduce the size of the overall design offering a simple and low-cost fabrication. Their use for diverse sensing applications such as chemical sensors [19,20], gas sensors [21,31], crack detection sensors [22,23], angle sensors [24], alignment and position sensors [25], displacement sensors [26] and microwave imaging sensors [27] has been already demonstrated.

In this paper, a microfluidic ethanol chemical sensor is proposed using the CSRR-loaded patch. The proposed sensor can detect the ethanol concentration from the frequency shift of the microfluidic CSRR. The microfluidic CSRR is designed in order to minimize the overall size and maximize the sensitivity. The design guideline of the CSRR and microfluidic channel is explained and the performance of the proposed sensor is demonstrated from simulation and measurement.

This paper is organized as follows: Section 2 explains the sensor design, Section 3 analyzes the sensitivity, and Section 4 gives details of the fabrication process and measurement results as well as a comparison with RF and non-RF sensors. Finally, we discuss the conclusion in Section 5.

## 2. Sensor Design

### 2.1. Design: CSRR-Loaded Patch

**Figure 1:**
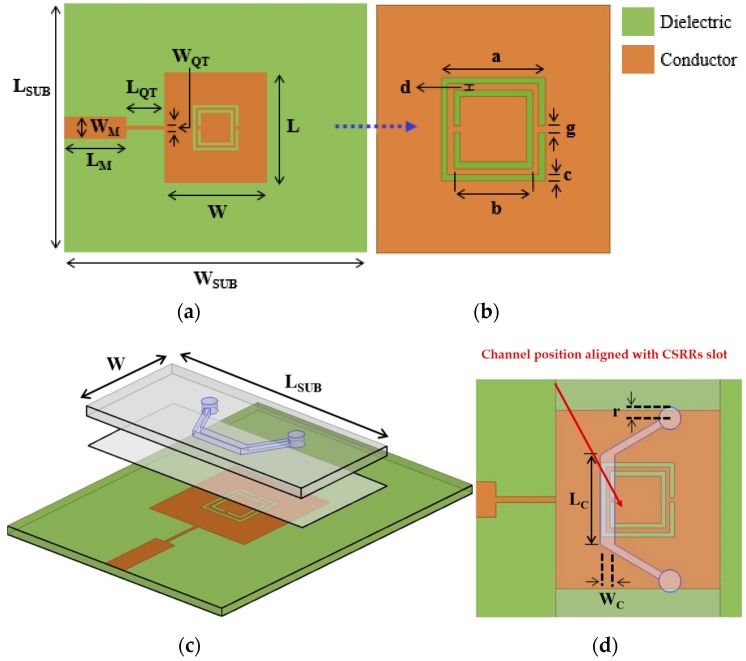
(**a**) Top view of CSRR-loaded patch fed by quarter-wave stub and microstrip line before loading PDMS; (**b**) Zoom-in image of the CSRRs slot; (**c**) Bird’s-eye view of the proposed sensor with the microfluidic channel; (**d**) Top view of channel alignment with CSRRs slot.

Figure 1 shows the layout of the CSRR-loaded rectangular patch fed by the quarter-wave stub and microstrip line. The Rogers RT/Duroid 5870 (Rogers Corporation, Connecticut, CT, USA) substrate (thickness = 0.78 mm, dielectric constant = 2.33, and loss tangent = 0.0012) is used to realize the design. The size of the patch (*L* × *W*) is 12 mm × 11.3 mm. The feed line is a combination of the microstrip line and quarter-wave transformer. The width of the microstrip line (*W*_M_ = 2.38 mm) is designed for the 50 Ω compatibility of the SMA (SubMiniature version A) (Sung-Jin Industrial Co., Ltd., Seoul, Korea) connector. The input impedance of the CSRR-loaded rectangular patch with the 50 Ω feed line is simulated using the ANSYS High Frequency Structural Simulator (HFSS) (ANSYS Inc., Canonsburg, PA, USA). After de-embedding the feed line, its impedance is 87.26 Ω at the resonant frequency. Therefore, the quarter-wave transformer with 66 Ω is used in order to match the impedance to 50 Ω. The length and width of the quarter-wave transformer (*L*_QT_ × *W*_QT_) is 4.1 mm and 0.4 mm, respectively, for a good impedance match. Two square-shaped CSRRs are loaded on the center of the patch such that the CSRR plane is perpendicular to the direction of propagation. The outer ring of the CSRR is designed with the side length (a), width (c), and split gap (g) as 5 mm, 0.3 mm, and 0.3 mm, respectively. The side length (b) of the inner ring is 3.8 mm, while the width and split gap are the same as those of the outer ring. The inter-distance (d) between the outer and inner rings of the CSRRs is investigated as 0.3 mm for critical coupling using parametric analysis on ANSYS HFSS. All geometrical parameters of our proposed sensor are mentioned in Table 1.

Before loading the CSRR, the length and width of the patch determine the resonant frequency. However, after introducing CSRRs, the side lengths, coupling gap and split gap of CSRRs are the parameters that control the resonant frequency of our proposed sensor.

**Figure 2:**
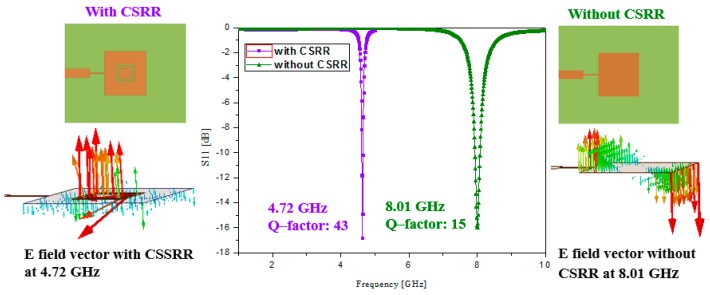
Comparison of resonant frequency and loaded Q-factor with and without CSRRs. E-field distributions with and without the CSRR are illustrated at 4.72 GHz and 8.01 GHz, respectively.

Figure 2 shows the simulated return losses of the patch with and without the CSRR. Before loading the CSRR, the proposed sensor resonates at 8.01 GHz. The inset of Figure 2 shows that the proposed sensor without the CSRR corresponds to the TM_010_ mode. After loading the CSRR, the proposed sensor resonates at 4.72 GHz. The inset of Figure 2 shows that the proposed sensor with the CSRR corresponds to the CSRR mode [30]. The resonant frequency at the CSRR mode is dependent on the geometrical parameters of the CSRR.

**Figure 3:**
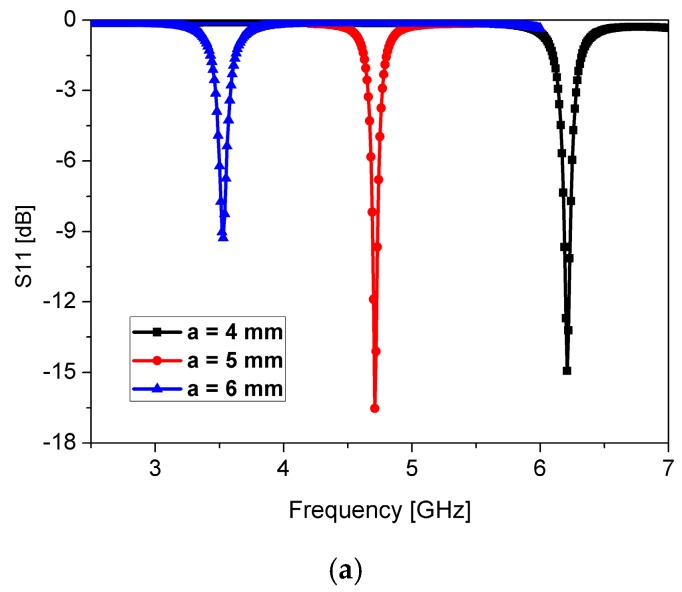

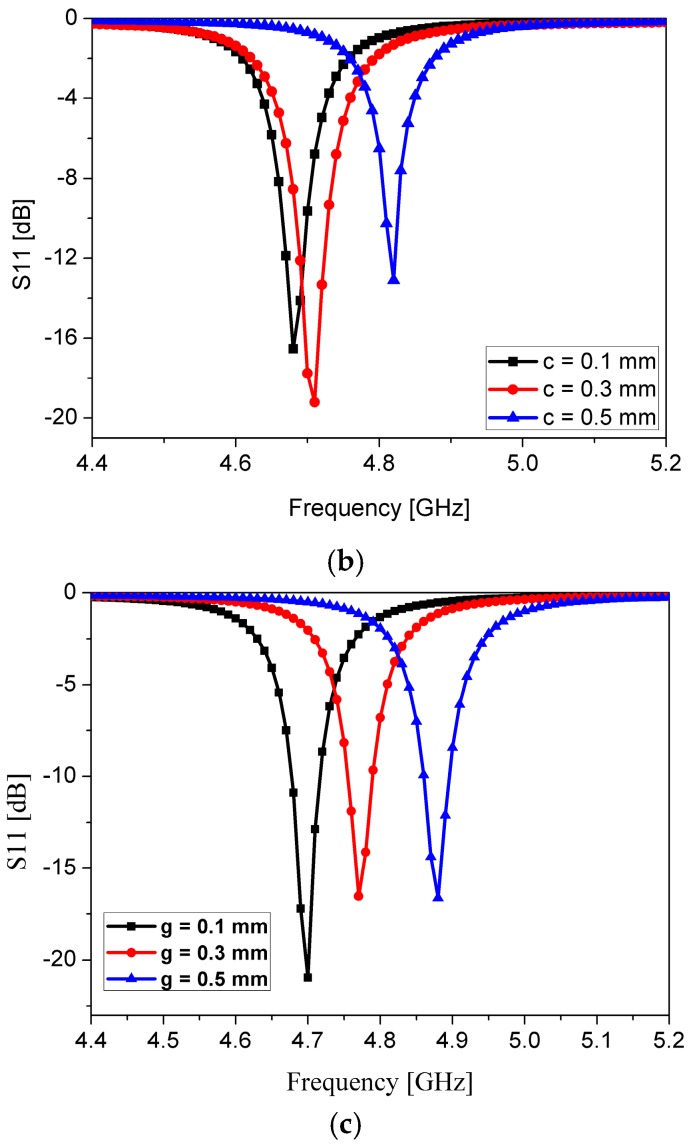
Simulated S_11_ of the CSRR-loaded patch resonator at different (**a**) slot length of *a*; (**b**) slot width of *c*; and (**c**) gap of *g*.

The fundamental formula of capacitance (*C*) is given as

(1) $$C=\frac{A\varepsilon_{\mathrm{eff}}}{d}$$

where ε_eff_ represents the effective permittivity seen by the capacitor, and A is the area of the metallic plates (surface of patch and ground plane) [32]. The slots of the CSRRs contain high E-field (electric field) energy and act similar to the way a cavity resonator works. Therefore, we can define the resonant frequency of the CSRR loaded patch as

(2) $$f=\frac{1}{2\pi \sqrt{L_{\mathrm{eff}}C_{\mathrm{eff}}}}$$

where *L*_eff_ and *C*_eff_ represent the effective inductance and capacitance of the resonator, respectively [33]. Figure 3 shows the simulated return losses when the slot length of *a*, slot width of *c*, and split gap of *g* are varied. When the slot length of *a* is increased, *L*_eff_ is increased. Therefore, the resonant frequency is decreased with longer *a* as shown in Figure 3a. It is observed from Figure 3b that the resonant frequency is increased with the wider *c* because of the lower *C*_eff_. When the split gap of *g* is increased, *C*_eff_ is decreased. Therefore, the resonant frequency is increased with a larger *g* as shown in Figure 3c. The final geometrical parameters of the proposed sensor in order to resonate at 4.72 GHz are listed in Table 1.

A high Q-factor, compact size, and efficient and inexpensive design are the prominent features of the proposed design, which we achieved because of the CSRRs. The simulated loaded Q-factor of the patch loaded by the CSRR (Q = 43) is roughly three times of that of the loaded Q-factor of the patch without introducing the CSRR (Q = 15), as shown in Figure 2. The loaded Q-factor is calculated from

(3) $$Q=\frac{f_{0}}{f_{3\mathrm{dB}}}$$

where *f*_0_ is the resonant frequency and *f*_3dB_ is the 3 dB bandwidth [33]; *f*_0_ and *f*_3dB_ are calculated from the S-parameters in Figure 2.

### 2.2. Microfluidic Channel Design

**Figure 4:**
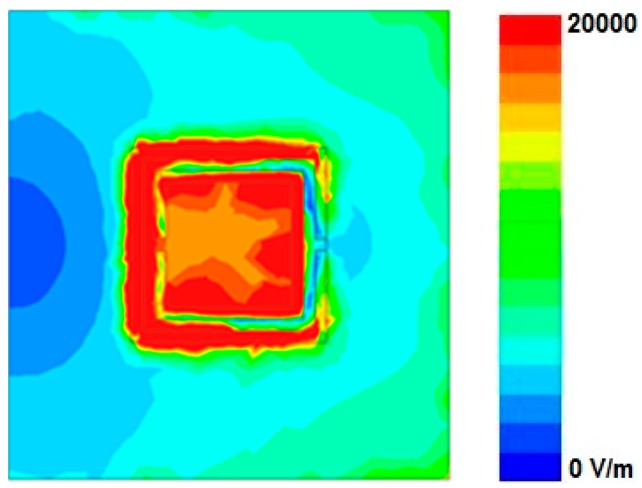
E-field distribution of CSRR-loaded patch at resonant frequency of 4.72 GHz (without microfluidic channel).

The microfluidic channel is designed after considering the maximum frequency shift, the fabrication limit, and the minimum alignment error. First of all, in order to decide the location of a microfluidic channel on the CSRR, the magnitude of the electric field distribution of the CSRRs loaded on a patch is plotted in Figure 4. To achieve the full benefits of the resonance originating from the CSRRs, it is important to identify the area of the highest sensitivity of the CSRR slot. The microfluidic channel should be accurately positioned on the most sensitive location in the design, so that strong interaction occurs between the electromagnetic field and the fluid injected in the channel.

**Figure 5:**
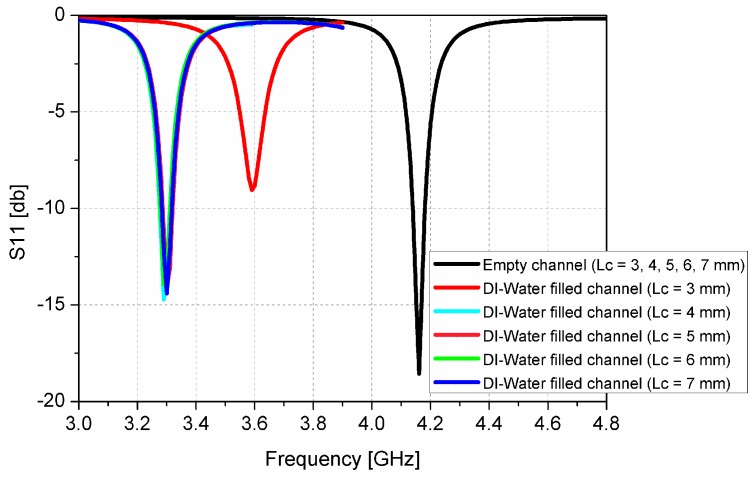
Simulated S_11_ of the proposed sensor with the empty and DI water-filled channel when *L*_C_ is varied from 3 mm to 7 mm.

When the deionized (DI) water inside the microfluidic channel is replaced by ethanol, the effective permittivity of the fluid changes, and this is used to tune the resonant frequency of the CSRRs. Figure 4 shows that a very high E-field exists around the CSRR slot. We observed that the left slot of the CSRRs is the most sensitive area of the dielectric substrate (indicated by the bright red color). Therefore, it is the best position to load the microfluidic channel. However, to achieve the best performance (that is, the maximum frequency shift) while consuming minimum fluid volumes, we investigated different lengths and widths of the channel. Due to fabrication limitations, the channel width (*W*_C_) and height (*H*_C_) are fixed as 1 mm and 0.6 mm, respectively. It is expected that the frequency shift between empty and chemical-filled channels is higher with a larger channel length (*L*_C_). In Figure 5, the simulated S_11_ of the proposed sensor with the empty and DI water-filled channel is shown. It is obvious that the resonant frequency with the empty channel is not changed, although *L*_C_ is varied from 3 mm to 7 mm. When *L*_C_ is varied from 3 mm to 7 mm, the resonant frequency with the DI water-filled channel is decreased and the frequency shift between the empty and chemical-filled channels is higher until *L*_C_ = 4 mm. In this work, we choose *L*_C_ as 5 mm after considering the alignment issue. Finally, an optimum geometry of the microfluidic channel (*L*_C_ × *W*_C_ × *H*_C_) is selected as 5 mm × 1 mm × 0.6 mm. The layout of the microfluidic channel integrated with the CSRR-loaded patch working as an ethanol sensor is shown in Figure 1c. To accurately align the position of the channel with the CSRR slots, the length and width of PDMS are decided to be the same as the length of the main substrate (*L*_SUB_ = 27 mm), and the width of the patch (*W* = 11.3 mm).

## 3. Sensitivity Analysis

**Figure 6:**
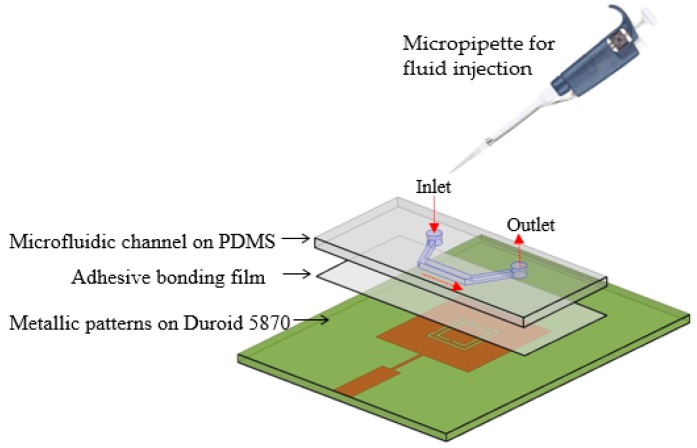
Side view of three layers of proposed CSRR-loaded patch as a microfluidic chemical sensor.

The resonant frequency of the CSRR is dependent on the permittivity of its surrounding materials. Therefore, when the liquid with different permittivity is loaded on the CSRR, the electric field is perturbed and the resonant frequency is changed. In this work, the microfluidic channel is introduced to inject the liquid as illustrated in Figure 6. In order to maximize the electric field perturbation, the microfluidic channel must be loaded where the strongest electric field is generated.

**Figure 7:**
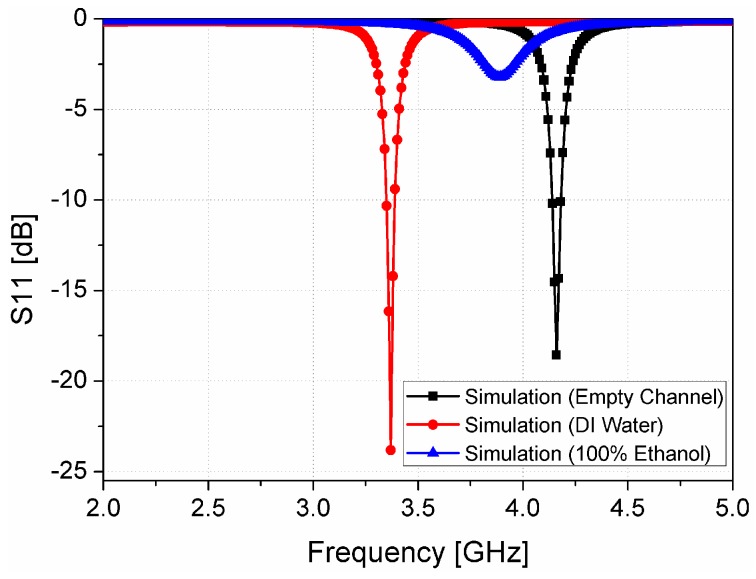
Simulated S-parameters of the proposed CSRR-loaded patch using empty channel (air), DI water, and 100% ethanol.

In order to quantify sensitivity, the resonant frequency of the empty channel and the DI water-filled channel (*f*_air_ and *f*_water_) are simulated. It is found that the relative change in the resonant frequency (Δ*f*_res_) is linearly related to the corresponding relative change in permittivity (Δε). The sensitivity S of our proposed sensor is defined as

(4) $$Sensitivity\;S\;=\frac{\Delta f_{\mathrm{res}}}{\Delta \varepsilon }=\left|\frac{f_{\mathrm{air}}-f_{\mathrm{water}}}{\varepsilon_{\mathrm{air}}-\varepsilon_{\mathrm{water}}}\right|$$

where *ε*_air_ is the permittivity of free space, and *ε*_water_ is the permittivity of deionized (DI) water. It is known that the dielectric constant and loss tangent values of DI water are 75–80 and 0.1, respectively [34,35,36]. The empty channel is widely used as a reference model because of its known permittivity and loss tangent of air. When a fluid is injected into the microfluidic channel, it exerts pressure on the air inside the channel and pushes it out through the hole. The fluid injected interacts with the sensitive part of the dielectric substrates, and causes it to switch the resonant frequency. The shift in resonant frequency is relative to the reference value and depends upon the permittivity of the fluid under test (ethanol in our case). To get a better insight in the sensitivity of the proposed sensor, the simulated S-parameters of our proposed sensor after loading the microfluidic channel are plotted in Figure 7. For full-wave simulation, the dielectric constant and tangential loss of PDMS are set as 2.8 and 0.05, respectively. The resonant frequency with the empty channel is 4.16 GHz while the resonant frequency without PDMS (see Figure 2) is 4.72 GHz. The frequency shifts from 4.16 GHz to 3.34 GHz, when the permittivity and loss tangent of the DI water are simulated. A frequency shift of 550 MHz is observed when ethanol is simulated in the channel, and the resonant frequency with ethanol is located as 3.89 GHz. The broader bandwidth of the proposed sensor with ethanol is due to the high dielectric loss of 100% ethanol. The dielectric constant and loss tangent of 100% ethanol for full-wave simulation are 6.5 and 0.4, respectively [37].

## 4. Fabrication and Measurement

The proposed sensor is realized using three different layers, as shown in Figure 6. The bottom layer consists of metallic patterns fabricated by chemical etching using conventional photolithography on the Rogers RT/Duroid 5870 substrate. The middle layer is a double-sided adhesive film with a 0.0508 mm thickness which serves the purpose of bonding between the Rogers DT/Duroid 5870 (Rogers Corporation, Connecticut, CT, USA) substrate and the PDMS substrate. In this work, ARcare^®^92848 manufactured by Adhesives Research, Inc. (Glen Rock, NJ, USA) is used for the adhesive film because it is used in biosensor spacers and general diagnostic device bonding [38]. Compared with the thickness of the PDMS (1 mm), the thickness of the adhesive film is much thinner. In addition, the dielectric constant of the PDMS and adhesive film are 2.8 and 3, respectively. Therefore, the adhesive film does not affect the frequency response of the proposed sensor which is also verified by full-wave simulation. A laser-etching machine is used to etch/engrave the microfluidic channel inside the PDMS substrate, and it serves as the top layer. The etching phenomenon of the laser-etching machine is a simple and quick process as compared to photolithography. Because the fixed position of the microfluidic channel is assured by same length of the Rogers and PDMS substrates, the sensor measurements showed reliability and excellent repeatability, which we verified after conducting more than 10 measurements with the same fabricated prototype sample and using the same measurement setup.

**Figure 8:**
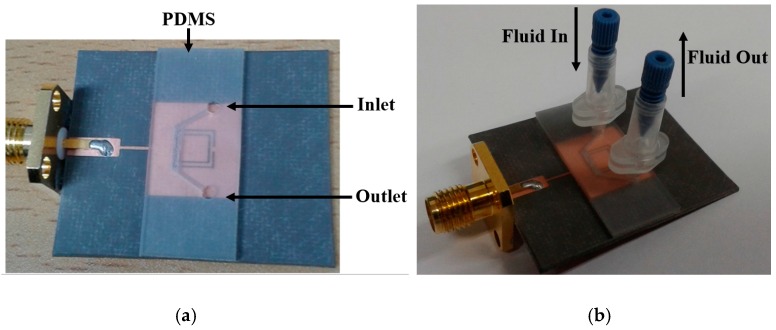
(**a**) Fabricated prototype of CSRR-loaded microfluidic patch as ethanol chemical sensor; (**b**) Side view with nanoport assembly.

To inject fluids in the channel, two holes are introduced inside the PDMS across the edges of the channel, and a nanoport assembly is employed as an inlet and outlet. In order to facilitate the precise injection/removal of the extremely small fluid (3 μL) in the channel, the NanoPort™ assembly (Coned Port Version) manufactured by IDEX corp. (Lake Forest, IL, USA) is used [39]. The height of these holes is 1 mm (same as the thickness of the PDMS), while their diameter is 1.5 mm after considering the tip of the coned nut. The fabricated prototype sample and the addition of the nanoport assembly on it are shown in Figure 8a,b, respectively. The ethanol sensing measurement was conducted by first injecting DI water to observe the reference resonant frequency. The microfluidic channel is cleaned by exerting air pressure through the micropipette, before introducing ethanol. The pipetman^®^ classic kit provided by Gilson Inc. (Middleton, WI, USA) is used for the micropipette [40]. The microfluidic channel is aligned with the slots of the CSRRs, and the effective permittivity of the dielectric is changed when the air inside the channel is replaced by liquid (DI water, and ethanol).

**Figure 9:**
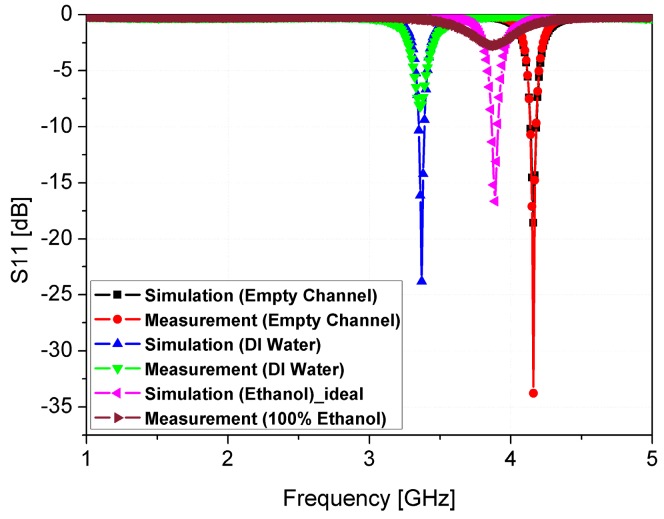
Comparison of simulated and measured S-parameters of the proposed microfluidic CSRR-loaded patch using air (empty channel), DI water, and 100% ethanol.

**Figure 10:**
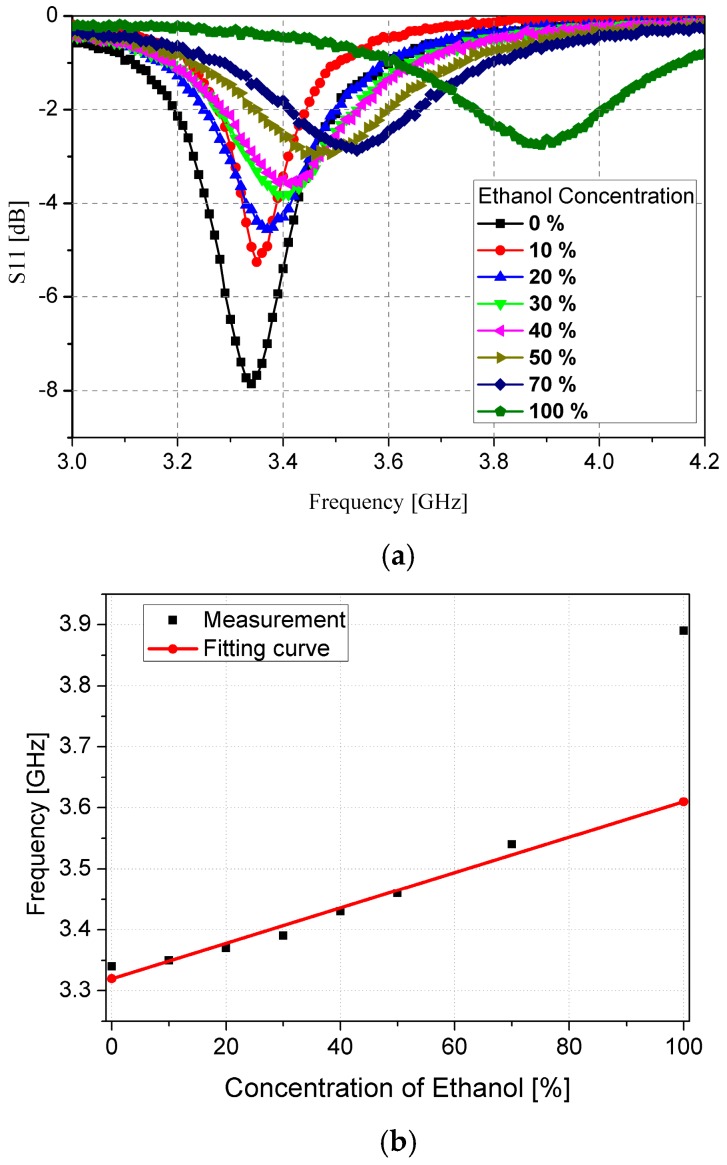
(**a**) Measured S-parameters; (**b**) Resonant frequency of ethanol with different concentrations, from 0% (DI water) to 100% with the fitting curve of *y* = 2.9 × 10^−3^ *x* + 3.32 in GHz.

In order to demonstrate the performance of the fabricated prototype of the ethanol chemical sensor, the S-parameters are measured using the Anritsu MS2038C network analyzer (Anritsu Corporation, Kanagawa Prefecture, Japan). The simulation and measurement results are compared and are in good agreement, as shown in Figure 9. The resonant frequency with an empty channel is observed at 4.16 GHz. The resonant frequency switches to 3.34 GHz and 3.89 GHz when DI water and pure ethanol are injected in the channel, respectively. In order to investigate the sensitivity of our proposed ethanol chemical sensor, the S-parameter values are measured at low concentrations of ethanol. Figure 10a shows the S-parameter of ethanol when the concentration is changed from 0% to 100%. The lower detection limit of our proposed ethanol chemical sensor is 10% relative to pure ethanol. Although this measurement is repeated five times, the resonant frequency is not changed and the limit of detection is not degraded. Therefore, the repeatability of the proposed sensor is successfully demonstrated. It is observed that the bandwidth becomes broader as the ethanol concentration increases because of the higher dielectric loss. The dielectric loss of 100% ethanol is higher than the dielectric loss of 0% ethanol (DI water). Therefore, the dielectric loss increases with the higher ethanol concentration. Figure 10b illustrates the range of resonant frequencies when the ethanol concentration varies from 0% to 100%. The relationship between the resonant frequency and the ethanol concentration is close to *y* = 2.9 × 10^−3^*x* + 3.32 from 0% to 70% concentrations. When the sensitivity of the ethanol sensor is defined by the slope angle of the fitting curve, it is 2.9 × 10^6^ Hz/percentage. These results successfully demonstrate and verify the utilization of our proposed CSRR-loaded patch as a microfluidic ethanol chemical sensor.

State-of-the-art sensors have achieved excellent sensitivity as reported by many research groups. They are using semiconductive thin films such as SnO_2_ [41,42], ZnO [43], TiO_2_ nanotubes [44], and carbon nanotubes (CNT) [45]. In the literature, it is claimed that recently published resistive electrochemical sensors have achieved ultra-high sensitivity for ethanol concentrations, such that their limit of detection ranges from 50 ppm to 5000 ppm [44]. Several techniques were proposed to overcome the shortcomings of these sensors, but they succeeded only partially. To overcome the poor selectivity, high cost, and high operating temperature problem, an outstanding ethanol gas sensor based on organic-inorganic hybrid composite sensing layers is proposed [46]. Despite its excellent performance, it suffers from complexity of the design process and fabrication. To sum up, sensing properties of metal oxides in these sensors are not independent, but rather are related to the preparation history, particle size, morphology and operating temperature [47]. The complexity involved in nanoparticle fabrication and the high costs are two other reasons that impede their wide acceptance.

The lower limit of detection of the proposed sensor is 10% ethanol. To prepare 10% ethanol, 1 mL of DI water is mixed with 100 μL of 100% ethanol. The absolute grade ethanol solvent (CH_3_CH_2_OH, part number 32,205) is imported from the SIGMA-ALDRICH Corporation (St. Louis, MO, USA). This solvent contains 789 g solute in 1 L of water [48], which means 10% ethanol corresponds to 78,990 ppm in our experiment. Nevertheless, the proposed sensor is miniaturized, non-contact, reusable, and requires extremely little fluid sample for testing. It is a low-cost sensor, and can be fabricated by traditional lithography (simple process).

The performance of the proposed sensor is compared with the performance of other RF sensors in Table 2. It is observed that the proposed sensor shows higher sensitivity and smaller electrical size compared with other RF sensors from [16,20,21,49]. In addition, the performance of the proposed RF sensor is compared with the performance of other non-RF sensors from [41,50,51,52,53] in Table 3. The proposed RF sensor shows lower sensitivity and higher limit of detection which are drawbacks of general RF sensors. Nevertheless, the RF sensor is non-contact and reusable. The simple fabrication process is the additional advantage of the proposed sensor.

## 5. Conclusions

In this paper, a CSRR-loaded microfluidic ethanol chemical sensor is proposed. The proposed ethanol chemical sensor is composed of two concentric CSRRs etched on top of a patch. A microfluidic channel is integrated on the most sensitive area of the CSRR slot. When a fluid is instilled in the microfluidic channel, the resonant frequency of the CSRRs is shifted because of the effective permittivity variation. The proposed ethanol chemical sensor is realized on Rogers/Duroid 5870 substrate using the photolithography process, while the microfluidic channel is engraved on PDMS using a laser-etching machine, which is a very quick process. The performance of our proposed sensor is validated using simulation and measurement results, and it shows a distinguishable frequency response when the concentration of ethanol is changed from 10% to 100%. Our proposed sensor is miniaturized, low-cost, non-contact, and reusable, which makes it a suitable candidate for monitoring low concentrations (up to 10%) of ethanol using extremely small volumes of liquid (only 3 μL) at room temperature. The reliability and repeatability of the proposed sensor are verified after conducing more than 10 measurements. For future consideration, we are planning to realize this sensor on a flexible substrate for its inclusion in wearable devices due to their growing demands. We also plan to test various chemicals to evaluate the selectivity of our sensor for medical applications.

## Figures and Tables

**Table 1:** Geometrical parameters of CSRR-loaded patch (unit: mm).

Parameter	Value	Parameter	Value	Parameter	Value	Parameter	Value
L	12	L_M_	6.75	c	0.3	d	0.3
W	11.3	W_M_	2.38	g	0.3	r	0.75
L_QT_	4.1	a	5	L_SUB_	27	W_SUB_	33
W_QT_	0.4	b	3.8				

**Table 2:** Comparison table of proposed sensor and other RF sensors.

References	Sensing Material	Frequency [GHz]	Frequency Shift (Δf) ^†^ [MHz]	Electrical Size ^††^	Sensitivity ^†^ (MHz/ε_r_)	Volume [μL]
[16]	Ethanol liquid	4.62–4.69	70	0.93λ_g_ × 0.81λ_g_	12.73	1
[20]	Ethanol liquid	4.23–4.48	250	1.26λ_g_ × 0.63λ_g_	45.45	5
[21]	Amonia gas	4.210–4.367	157	1.29λ_g_ × 1.29λ_g_	N/A	N/A
[49]	Ethanol gas	14.85–15.07	220	2.71λ_g_ × 1.35λ_g_	40	N/A
This Work	Ethanol liquid	3.89–4.16	270	0.63λ_g_ × 0.51λ_g_	49.1	3

^†^ Frequency shift (Δ*f*) and sensitivity (Δ*f*/Δε_r_) are estimated from air and ethanol; ε_r_ of ethanol is assumed to be 6.5; ^††^ λ_g_ is guided wavelength.

**Table 3:** Comparison table of proposed sensor and non-RF sensors.

References	Sensing Materials	Contact	Fabrication Complexity	Sensitivity	Limit of Detection
[41]	Ethanol gas	contact	Complex	31.4 ^†^	10 ppm @ 300 °C
[50]	Ethanol liquid	contact	Complex	57.3 ^†^	100 ppm @ 400 °C
[51]	Ethanol vapor	contact	Complex	4.6 ^†^	300 ppm @ 350 °C
[52]	Ethanol gas	contact	Complex	24.5 ^†^	50 ppm @ 25 °C
[53]	Ethanol vapor	contact	Complex	19.6 ^†^	10 ppm @ 220 °C
This Work	Ethanol liquid	Non-contact	Simple	49.1 ^††^ MHz/ε_r_	78,990 ppm

^†^ Sensitivity is defined as a ratio of resistance of air and gas (R_air_/R_gas_); ^††^ Sensitivity is defined as a Δ*f*/Δε_r_.
